# Bioconversion of D-galacturonate to keto-deoxy-L-galactonate (3-deoxy-L-*threo*-hex-2-ulosonate) using filamentous fungi

**DOI:** 10.1186/1472-6750-10-63

**Published:** 2010-08-26

**Authors:** Marilyn G Wiebe, Dominik Mojzita, Satu Hilditch, Laura Ruohonen, Merja Penttilä

**Affiliations:** 1 VTT Technical Research Centre of Finland, P.O. Box 1000, FI-02044 VTT, Finland

## Abstract

**Background:**

The D-galacturonic acid derived from plant pectin can be converted into a variety of other chemicals which have potential use as chelators, clarifiers, preservatives and plastic precursors. Among these is the deoxy-keto acid derived from L-galactonic acid, keto-deoxy-L-galactonic acid or 3-deoxy-L-*threo*-hex-2-ulosonic acid. The keto-deoxy sugars have been found to be useful precursors for producing further derivatives. Keto-deoxy-L-galactonate is a natural intermediate in the fungal D-galacturonate metabolic pathway, and thus keto-deoxy-L-galactonate can be produced in a simple biological conversion.

**Results:**

Keto-deoxy-L-galactonate (3-deoxy-L-*threo*-hex-2-ulosonate) accumulated in the culture supernatant when *Trichoderma reesei *Δ*lga1 *and *Aspergillus niger *Δ*gaaC *were grown in the presence of D-galacturonate. Keto-deoxy-L-galactonate accumulated even if no metabolisable carbon source was present in the culture supernatant, but was enhanced when D-xylose was provided as a carbon and energy source. Up to 10.5 g keto-deoxy-L-galactonate l^-1 ^was produced from 20 g D-galacturonate l^-1 ^and *A. niger *Δ*gaaC *produced 15.0 g keto-deoxy-L-galactonate l^-1 ^from 20 g polygalacturonate l^-1^, at yields of 0.4 to 1.0 g keto-deoxy-L-galactonate [g D-galacturonate consumed]^-1^. Keto-deoxy-L-galactonate accumulated to concentrations of 12 to 16 g l^-1 ^intracellularly in both producing organisms. This intracellular concentration was sustained throughout production in *A. niger *Δ*gaaC*, but decreased in *T. reesei*.

**Conclusions:**

Bioconversion of D-galacturonate to keto-deoxy-L-galactonate was achieved with both *A. niger *Δ*gaaC *and *T. reesei *Δ*lga1*, although production (titre, volumetric and specific rates) was better with *A. niger *than *T. reesei*. *A. niger *was also able to produce keto-deoxy-L-galactonate directly from pectin or polygalacturonate demonstrating the feasibility of simultaneous hydrolysis and bioconversion. Although keto-deoxy-L-galactonate accumulated intracellularly, concentrations above ~12 g l^-1 ^were exported to the culture supernatant. Lysis may have contributed to the release of keto-deoxy-L-galactonate from *T. reesei *mycelia.

## Background

Cellulose, hemicellulose, lignin and pectin are among the most abundant carbon reserves on earth, all present in plant biomass. While cellulose, hemicellulose and lignin are particularly abundant in grasses and woody plants, pectin is abundant in many fruits and some roots, such as the sugar beet (*Beta vulgaris *L). Pectin may be purified and used as a gelling agent and stabilizer, for instance in the food and pharmaceutical industries, or may be hydrolysed to release monomers, primarily D-galacturonic acid, which find limited use as chelating agents. D-Galacturonic acid may be electrolytically oxidised to galactaric (mucic) acid, avoiding the high concentrations of nitrous oxide which are used to produce galactaric acid from D-galactose or lactose [1]. Galactaric acid is used in similar applications to D-galacturonic acid, but may also be used in modifying plastics [2]. In addition to being oxidised to mucic acid, D-galacturonic acid can also be reduced to L-galactonic acid [3], for applications similar to those with galactaric and D-galacturonic acids. The range of applications for these acids continues to expand.

Interest in galactonic acid derivatives has increased on account of the planar zigzag conformation they can adopt in solution [e.g. [2,4]], while keto sugars are useful intermediates in the production of various sugar derivatives [5,6]. Removal of water from L-galactonic acid leads to the formation of keto-deoxy-L-galactonic acid (3-deoxy-L-*threo*-hex-2-ulosonic acid). Keto-deoxy sugars have potential as precursors in the synthesis of medicinal and other compounds [7]. Keto-deoxy-L-galactonate is an intermediate in the metabolism of D-galacturonate by fungi [8] and the genes encoding D-galacturonate reductase (*gar1 *&*gaaA*), L-galactonate dehydratase (*lgd1 *&*gaaB*) and 2-keto-3-deoxy-L-galactonate aldolase (*lga1 *&*gaaC*) have been identified in *Trichoderma reesei *(anamorph of *Hypocrea jecorina*) [3,9,10] and *Aspergillus niger *[11]. Deletion of any one of these three genes in *T. reesei *results in a strain unable to grow on D-galacturonate as sole carbon source. In this paper we describe the conversion of D-galacturonate to 2-keto-3-deoxy-L-galactonate using strains of *T. reesei *and *A. niger *from which the 2-keto-3-deoxy-L-galactonate aldolase encoding gene (*lga1 *and *gaaC*, respectively) has been deleted.

## Results

### Bio-conversion of D-galacturonate to keto-deoxy-L-galactonate with no added energy source

**Figure 1:**
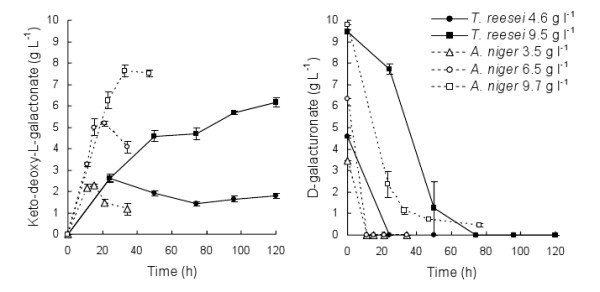
**Bio-conversion of D-galacturonate to keto-deoxy-L-galactonate**. Conversion of D-galacturonate to keto-deoxy-L-galactonate by *T. reesei *Δ*lga1 *(solid symbols) and *A. niger *Δ*gaaC *(open symbols) in flasks at 30°C, 200 rpm. Mycelia were pre-grown in medium containing 20 g D-xylose and 1 g peptone l^-1^, and were washed with sterile H_2_O before incubation in D-galacturonate, initial pH 5.2 to 5.5. D-galacturonate concentration is shown on the right. Error bars represent ± standard error of the mean.

*T. reesei *Δ*lga1 *converted D-galacturonate to keto-deoxy-L-galactonate at an initial rate of 0.10 ± 0.01 g keto-deoxy-L-galactonate l^-1 ^h^-1 ^(~0.03 g [g biomass]^-1 ^h^-1^) When 4.6 g D-galacturonate was provided, 2.6 g keto-deoxy-L-galactonate l^-1 ^was produced in the culture supernatant within 24 h (Figure 1). However, product was subsequently degraded or removed from the solution through an unknown mechanism. In 9.5 g D-galacturonate l^-1^, 6.2 ± 0.2 g keto-deoxy-L-galactonate l^-1 ^was produced (yield = 0.6 g g^-1^) and degradation was not observed. When *T. reesei *Δ*lga1 *was grown in bioreactors, the degradation of keto-deoxy-L-galactonate did not result in increased biomass production or release of measureable amounts of CO_2_.

*A. niger *Δ*gaaC *was incubated in 3.5 to 9.7 g D-galacturonate l^-1 ^in flasks to assess its ability to convert D-galacturonate to keto-deoxy-L-galactonate (Figure 1). *A. niger *Δ*gaaC *produced more keto-deoxy-L-galactonate (7.6 ± 0.3 g keto-deoxy-L-galactonate l^-1 ^from 9.8 g D-galacturonate l^-1^) at a higher rate (0.27 ± 0.02 g keto-deoxy-L-galactonate l^-1 ^h^-1^, 0.13 ± 0.00 g [g biomass]^-1 ^h^-1^) than *T. reesei *Δ*lga1 *(Figure 1). The yield of keto-deoxy-L-galactonate on D-galacturonate was 0.96 ± 0.04 and 0.88 ± 0.02 g g^-1 ^in 6.3 and 10 g D-galacturonate l^-1^, respectively. Degradation of keto-deoxy-L-galactonate was again observed (Figure 1), with no measureable increase in biomass. In a pH regulated bioreactor (pH 5.6) containing ~2 g biomass l^-1 ^in 20 g D-galacturonate l^-1 ^solution 10.6 g keto-deoxy-L-galactonate was produced. The initial production rate was 0.20 g keto-deoxy-L-galactonate l^-1 ^h^-1 ^(~0.11 g [g biomass]^-1 ^h^-1^) and the yield ~1 g keto-deoxy-L-galactonate [g D-galacturonate consumed]^-1^.

The pH of the supernatant increased to between 7.5 and 8.0 when *T. reesei *Δ*lga1 *and *A. niger *Δ*gaaC *were incubated in unbuffered D-galacturonate solutions in flasks. Viable hyphae were still present during incubation in D-galacturonate solutions and *T. reesei *Δ*lga1 *showed normal sporulation. The vital stain methylene blue was used for a qualitative assessment of cell vitality. Considerable staining and cell shearing were observed in both control and keto-deoxy-L-galactonate producing strains incubated in the same conditions.

### The effect of added D-xylose on the bio-conversion of D-galacturonate to keto-deoxy-L-galactonate

**Figure 2:**
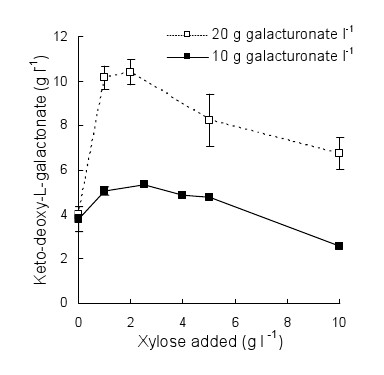
**Keto-deoxy-L-galactonate production in media supplemented with D-xylose**. Keto-deoxy-L-galactonate produced by *T. reesei *Δ*lga1 *in flasks (200 rpm) or bioreactors (pH 5.5, 500-600 rpm, 1 vvm air) at 30°C. Mycelia were pre-grown in medium containing 20 g D-xylose and 1 g peptone l^-1^, and were washed with sterile H_2_O before incubation in medium containing approximately 10 (solid symbols) or 20 (open symbols) g D-galacturonate l^-1 ^with 0 to 10 g D-xylose l^-1^. Error bars represent ± standard error of the mean.

Conversion of D-galacturonate to keto-deoxy-L-galactonate is an NADPH-requiring process. Although pre-grown mycelium has some NADPH, conversion of D-galacturonate to keto-deoxy-L-galactonate could be more efficient if a co-substrate was provided as an energy source to replenish NADPH. Addition of 1 to 10 g D-xylose l^-1 ^(in the presence of mineral salts and trace elements) improved (p < 0.05) the initial rate of conversion of D-galacturonate to keto-deoxy-L-galactonate by *T. reesei *Δ*lga1 *from 0.10 to 0.14 ± 0.004 g keto-deoxy-L-galactonate l^-1 ^h^-1^. The highest concentrations of keto-deoxy-L-galactonate from D-galacturonate were observed with 1 or 2 g D-xylose l^-1^, with 10.4 ± 0.6 g keto-deoxy-L-galactonate l^-1 ^being produced in medium containing 19 g D-galacturonate l^-1 ^and 2 g D-xylose l^-1^, at a yield of 0.55 g [g substrate]^-1 ^(Figure 2). With these concentrations of D-xylose, the specific production rate was similar to that in D-galacturonate solution without D-xylose (~0.03 g keto-deoxy-L-galactonate [g biomass]^-1 ^h^-1^), but with higher concentrations of D-xylose (5 or 10 g D-xylose l^-1^) specific production was reduced to ~0.01 g keto-deoxy-L-galactonate [g biomass]^-1 ^h^-1^. Higher specific production rates were observed in pH-controlled bioreactor cultures (~0.06 g [g biomass]^-1 ^h^-1^) than in flask cultures.

Incubation of *A. niger *Δ*gaaC *in medium containing 9.7 g D-galacturonate l^-1 ^and 2 g D-xylose l^-1 ^resulted in the production of 7.7 ± 0.5 g keto-deoxy-L-galactonate l^-1 ^at a rate of 0.33 ± 0.01 g l^-1 ^h^-1 ^(0.12 ± 0.02 g [g biomass]^-1 ^h^-1^) and a yield of 0.85 ± 0.05 g [g D-galacturonate consumed]^-1^. The pH after 76 h incubation had decreased to 2.8.

D-galacturonate was taken up at 0.19 ± 0.01 g D-galacturonate l^-1 ^h^-1 ^(~0.06 g D-galacturonate [g biomass]^-1 ^h^-1^) by *T. reesei *Δ*lga1 *and 0.28 ± 0.01 g D-galacturonate l^-1 ^h^-1 ^(~0.10 g D-galacturonate [g biomass]^-1 ^h^-1^) by *A. niger *Δ*gaaC *in flask cultures at low biomass concentrations, with or without added xylose. Higher volumetric uptake rates (up to 0.38 g D-galacturonate l^-1 ^and 0.56 g D-galacturonate l^-1 ^for *T. reesei *Δ*lga1 *and *A. niger *Δ*gaaC*, respectively), but lower specific uptake rates (~0.04 to 0.06 g [g biomass]^-1 ^h^-1^), were observed for both strains with higher biomass concentrations. In bioreactor cultures D-galacturonate uptake rates were lower than in flasks and similar (0.12 g D-galacturonate l^-1 ^h^-1^; ~0.06 g D-galacturonate [g biomass]^-1 ^h^-1^) for the two strains.

### Intracellular concentrations of keto-deoxy-L-galactonate

**Figure 3:**
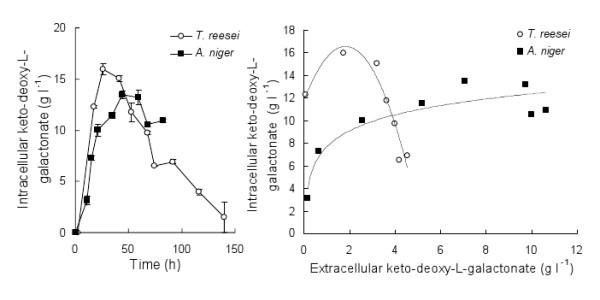
**Intracellular keto-deoxy-L-galactonate concentrations**. Intracellular concentrations of keto-deoxy-L-galactonate (left) produced by *A. niger *Δ*gaaC *(solid symbols) and *T. reesei *Δ*lga1 *(open symbols) when maintained in bioreactors at constant pH 5.5 on 17-19 g D-galacturonate l^-1 ^at 30°C, and the correlation to extracellular keto-deoxy-L-galactonate concentration (right) when D-galacturonate was still present in the culture supernatant. 4.2 g D-xylose l^-1 ^and mineral salts were added to the *T. reesei *culture but not to the *A. niger *culture. Cultures contained approximately 2 g biomass l^-1^. Error bars represent ± SEM and lines indicate trends in the data.

Keto-deoxy-L-galactonate was detected intracellularly within 17 h (12.3 ± 0.1 g l^-1^) incubation in medium containing 16.8 g D-galacturonate l^-1 ^and 4.2 g D-xylose l^-1 ^in *T. reesei *Δ*lga1 *(~2 g biomass l^-1^) and within 11 h (3.1 ± 0.5 g l^-1^) incubation in 19 g D-galacturonate l^-1 ^in *A. niger *Δ*gaaC *(~2 g biomass l^-1^), when extracellular keto-deoxy-L-galactonate was only 0.07 to 0.15 g l^-1 ^(Figure 3). In *T. reesei*, the intracellular keto-deoxy-L-galactonate concentration increased to 15.9 ± 0.5 g keto-deoxy-L-galactonate l^-1 ^and then decreased at an almost linear rate (0.13 g l^-1 ^h^-1^; Figure 3) as the extracellular concentration increased. In *A. niger *a maximum intracellular keto-deoxy-L-galactonate concentration of 13.5 ± 0.4 g l^-1 ^was observed after 44 h incubation, but the intracellular concentration was approximately constant (11.6 ± 0.6 g l^-1^) from 21 to 82 h (Figure 3), as extracellular concentrations increased. Biomass concentration decreased in both *A. niger *and *T. reesei *cultures at similar rates, reflecting attachment of biomass to surfaces in the bioreactor and/or cell lysis. Cell lysis, however, would have contributed at most 0.2 g keto-deoxy-L-galactonate l^-1 ^to the supernatant of the *T. reesei *culture and less than 0.1 g l^-1 ^to the supernatant of the *A. niger *culture.

### Conversion of polygalacturonate and pectin to keto-deoxy-L-galactonate

**Figure 4:**
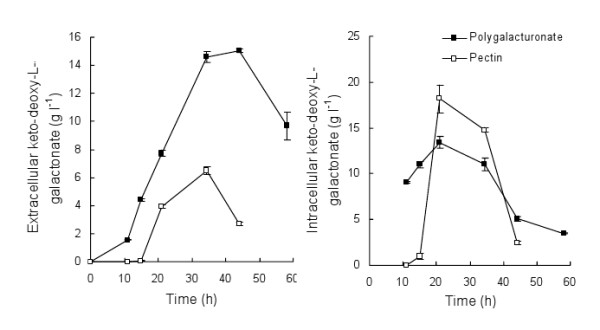
**Bio-conversion of pectin and polygalacturonate to keto-deoxy-L-galactonate**. Extracellular (left) and intracellular (right) keto-deoxy-L-galactonate production by *A. niger *Δ*gaaC *from 20 g l^-1 ^polygalacturonate (solid symbols) or pectin (open symbols). Media (pH ~5.3) containing 2 g D-xylose l^-1 ^and 20 g polygalacturonate l^-1 ^or 2.9 g D-xylose l^-1 ^and 20 g pectin l^-1 ^were inoculated with mycelia (~4.7 g biomass l^-1^) and incubated in flasks at 30°C, 200 rpm.

*A. niger *Δ*gaaC *produced keto-deoxy-L-galactonate directly from polygalacturonate and from pectin (Figure 4). 6.5 ± 0.3 g keto-deoxy-L-galactonate l^-1 ^was produced from 20 g pectin l^-1 ^(yield ~0.7 g keto-deoxy-L-galactonate [g D-galacturonate]^-1^) and 15.0 ± 0.1 g keto-deoxy-L-galactonate l^-1 ^from 20 g polygalacturonate l^-1 ^(yield ~1 g keto-deoxy-L-galactonate [g D-galacturonate]^-1^), at rates of 0.31 ± 0.02 g l^-1 ^h^-1 ^and 0.54 ± 0.2 g l^-1 ^h^-1^, respectively. Maximum intracellular keto-deoxy-L-galactonate (13 to 18 g keto-deoxy-L-galactonate l^-1^, Figure 4) was observed at 21 h in both pectin and polygalacturonate. Degradation of extracellular keto-deoxy-L-galactonate was observed at the end of the cultivations.

Hydrolysis of polygalacturonate was rapid, with 3.8 g D-galacturonate l^-1 ^present in the supernatant after only 11 h and increasing to 10.0 g D-galacturonate l^-1 ^at 15 h, after which concentrations decreased. D-galacturonate did not accumulate intracellularly, or was present in very low amounts. D-galacturonate (2.3 ± 0.1 g D-galacturonate l^-1^) was not observed in the culture supernatant of the pectin cultures until 21 h, when 3.9 g keto-deoxy-L-galactonate l^-1 ^had already been produced. After 21 h the concentration of D-galacturonate in the supernatant decreased. Intracellular D-galacturonate was observed in these cultures (~5 g l^-1^) and was detectable after only 15 h.

In addition to keto-deoxy-L-galactonate, approximately 6 g biomass l^-1 ^was produced in 20 g pectin l^-1 ^and 2 g biomass l^-1 ^in 20 g polygalacturonate l^-1^, of which 1-1.5 g biomass l^-1 ^would have been derived from the 2-3 g D-xylose l^-1 ^provided to the cultures and the rest from the carbohydrates present in the pectin or polygalacturonate. Growth resulted in a decrease in pH in both media, to 4.4 in polygalacturonate and 2.7 in pectin.

In contrast, *T. reesei *Δ*lga1 *did not produce keto-deoxy-L-galactonate when incubated in 10 g pectin l^-1 ^and 1.5 g D-xylose l^-1^. D-Xylose was consumed and ~0.8 g biomass l^-1 ^was produced, but no D-galacturonate was detected in the culture supernatant.

## Discussion

Hilditch *et al*. [10] observed the conversion of D-galacturonate to keto-deoxy-L-galactonate when conidia of *T. reesei *Δ*lga1 *were incubated in medium containing 0.5 g peptone l^-1 ^and 20 g D-galacturonate l^-1^. The peptone allowed conidial germination, resulting in 0.2 g biomass l^-1 ^and keto-deoxy-L-galactonate was produced extracellularly at a rate of approximately 0.025 g l^-1 ^h^-1^. Here we demonstrate that the conversion rate is substantially increased to 0.10 g l^-1 ^h^-1 ^when cultures are inoculated with more biomass and that the rate can be further increased (0.14 g l^-1 ^h^-1^) by including a carbon/energy source (up to 2 g D-xylose l^-1^) in the medium. Keto-deoxy-L-galactonate was produced after D-xylose had been consumed and high concentrations of D-xylose limited production.

*A. niger *Δ*gaaC *was more efficient than *T. reesei *Δ*lga1 *at producing keto-deoxy-L-galactonate, with production rates of 0.27 to 0.33 g l^-1 ^h^-1^, in solutions without and with added D-xylose, respectively. *A. niger *Δ*gaaC *also produced keto-deoxy-L-galactonate directly from citrus peel pectin or polygalacturonate at similar or higher (0.54 g l^-1 ^h^-1^) rates. These rates were comparable or higher to those demonstrated for the production of 2-keto-D-galactonate from D-galactose (approx. 0.14 g l^-1 ^h^-1^) by *Pseudomonas fluorescens *[5] and considerably higher than those observed for 2-keto-L-galactonate production by *Erwinia herbicola *(<0.2 mg l^-1 ^h^-1^; [12].

D-galacturonate uptake rates were generally higher in *A. niger *Δ*gaaC *than in *T. reesei *Δ*lga1*, which would contribute to the higher keto-deoxy-L-galactonate production rate in *A. niger*, but not the titre or yield. Further, the two strains had similar uptake rates in bioreactor cultures, but *A. niger *Δ*gaaC *still produced keto-deoxy-L-galactonate at a higher rate than *T. reesei *Δ*lga1*. The low rate of D-galacturonate uptake in bioreactor, compared to flask culture, was surprising, but may reflect differences in morphology in flasks and bioreactors, particularly for *A. niger *Δ*gaaC*. Little is known about D-galacturonate transporters in filamentous fungi, but putative transporters have recently been identified in *A. niger *[11] and two of these have homologues in *T. reesei *(unpublished result), although their function has not been confirmed. *T. reesei *appears to have a capacity, similar to or only slightly lower than that of *A. niger*, to take up D-galacturonate, but this capacity is not always realised, as also observed during galactarate production [13].

Measurement of intracellular keto-deoxy-L-galactonate concentrations in both *T. reesei *and *A. niger *demonstrated that it accumulated inside the cells to approximately 12 to 16 g l^-1^, and was detectable intracellularly before being detectable in the supernatant, suggesting that export of keto-deoxy-L-galactonate at pH 5.5 may be limited. In contrast, citrate is reported to accumulate intracellularly to only 0.4 to 6 g citrate l^-1 ^in *A. niger *when producing citrate [14]. None-the-less, intracellular accumulation of keto-deoxy-L-galactonate to 12 - 16 g l^-1 ^did not appear to strongly affect hyphal viability or ability of *T. reesei *to sporulate. In *A. niger*, 12 g intracellular keto-deoxy-L-galactonate l^-1 ^appeared to be a threshold above which all keto-deoxy-L-galactonate was exported to the supernatant (Figure 3). In *T. reesei*, the intracellular concentration decreased as extracellular concentrations increased (Figure 3), suggesting that *T. reesei *was able to induce an efficient export system, or that cell lysis contributed to its release into the supernatant. Release of keto-deoxy-L-galactonate from the cytoplasm would have contributed less than 0.2 g keto-deoxy-L-galactonate l^-1 ^in the supernatant and the continued uptake and conversion of D-galacturonate demonstrated that cell lysis alone was not responsible for the export in *T. reesei*. Export of organic acids by fungi is not well understood, but often involves active transport, as in citrate export by *A. niger *[14] or sorbate export by *S. cerevisiae *[15]. If export of keto-deoxy-L-galactonate requires active transport, the separation of growth and production phases would be undesirable and a substrate limited fed-batch process may enable higher over-all production rates. Transporters specific for keto-deoxy-L-galactonate would not be expected in either *A. niger *or *T. reesei*, which would not normally accumulate keto-deoxy-L-galactonate intracellularly, however other transporters might be expected to have various levels of affinity to transport keto-deoxy-L-galactonate in addition to their target molecules.

The theoretical yield of keto-deoxy-L-galactonate from D-galacturonate is 0.92 g g^-1 ^(1 mol mol^-1^). *T. reesei *Δ*lga1 *converted D-galacturonate to keto-deoxy-L-galactonate with an average yield of 0.5 g g^-1 ^in flask cultures and up to 0.74 g g^-1 ^in pH controlled bioreactor cultures, although the yield tended to decrease as the cultures progressed. D-Galacturonate did not accumulate in the cytoplasm in *T. reesei*, so uptake and storage of D-galacturonate was not responsible for the low yield. Keto-deoxy-L-galactonate concentrations decreased in some cultures, indicating that it could be degraded into non-metabolisable products or complex with other organic molecules.

The yield of keto-deoxy-L-galactonate from D-galacturonate was higher with *A. niger *Δ*gaaC *(0.7 to approximately 1.0) than with *T. reesei *Δ*lga1*, the theoretical yield being achieved in pure D-galacturonate solution and from polygalacturonate. Values above 0.92 g g^-1 ^reflect the difficulty of measuring D-galacturonate accurately when keto-deoxy-L-galactonate was present. Keto-deoxy-L-galactonate degradation was also observed in *A. niger *Δ*gaaC *cultures. Futile metabolism of D-galacturonate has previously been observed in *T. reesei *Δ*gar1 *and *A. niger *Δ*gaaA *strains, with or without co-expression of a uronate dehydrogenase [13].

*A. niger *Δ*gaaC *produced more keto-deoxy-L-galactonate than *T. reesei *Δ*lga1*, at higher rates. The *A. niger *strain chosen as producer (ATCC1015) is a known citric acid producer and is more acid tolerant than *T. reesei*. Both low pH tolerance and high citrate production suggest that *A. niger *ATCC1015 has more efficient acid export than *T. reesei*, which could contribute to improved production. However, differences in the D-galacturonate reductases (encoded by *gaaA *and *gar1*) and L-galactonate dehydratases (encoded by *gaaB *and *lgd1*) may also contribute to the higher production in *A. niger*, as would differences in D-galacturonate uptake in some conditions. Further, *A. niger *produced sufficient pectinases to produce keto-deoxy-L-galactonate directly from pectin or polygalacturonate, whereas *T. reesei *did not, as expected given the limited pectinase encoding genes in its genome [16]. Simultaneous hydrolysis and bioconversion is thus feasible with *A. niger *Δ*gaaC*, and the non-galacturonate sugars in pectin could provide the co-substrate for biomass and energy production.

## Conclusions

*A. niger *Δ*gaaC *and *T. reesei *Δ*lga1 *convert D-galacturonate to 2-keto-3-deoxy-L-galactonate, which accumulates extracellularly. Production rates were comparable or better than those observed for similar products with bacterial cells [5,12] and could be further improved by optimising the provision of co-substrate for biomass and energy production. Total production, volumetric production rate and specific production rate were higher for *A. niger *Δ*gaaC *than *T. reesei *Δ*lga1 *and the ability of *A. niger *Δ*gaaC *to produce keto-deoxy-L-galactonate directly from pectin or polygalacturonate demonstrate that it is the more desirable production organism.

Although keto-deoxy-L-galactonate accumulated intracellularly, concentrations above ~12 g l^-1 ^were exported to the culture supernatant. Lysis may have contributed to the release of keto-deoxy-L-galactonate from *T. reesei *mycelia. Since keto-deoxy-L-galactonate was not stable in the culture supernatant, it would be necessary to closely monitor a production process to obtain maximum yields and it would be advantageous to determine the path by which the keto-deoxy-L-galactonate is destroyed.

## Methods

### Strains

*Trichoderma reesei *(teleomorph *Hypocrea jecorina*) QM6a Δ*lga1*, lacking the gene encoding 2-keto-3-deoxy-L-galactonate aldolase, was generated as described in [10]. Stock cultures were maintained as conidia suspended in 20% v/v glycerol, 0.8% w/v NaCl with ~0.025% v/v Tween 20 at -80°C.

The cassette for deletion of *gaaC *from *A. niger *ATCC1015 contained 1971 bp from the *A. niger gaaC *promoter, 1693 bp from the *A. niger gaaC *terminator, and a 1927 bp fragment containing the *pyrG *gene flanked with its native promoter and terminator. These fragments were obtained by PCR from *A. niger *ATCC1015 genomic DNA using primers gaaC-5-F, gaaC-5-R, gaaC-3-F, gaaC-3-R, pyrG-del-F_n, and pyrG-del-R_n (Table 1), and the proofreading DNA polymerase Phusion (Finnzymes). Plasmid pRSET-A (Invitrogen) was digested with *Nhe*I (NEB) and *Ecl*136II (Fermentas), and the promoter fragment (*gaaC-5*) with *Nhe*I, to produce an intermediary construct by ligation using T4 DNA ligase (NEB). This intermediary construct and the terminator fragment (*gaaC-3*) were digested with *Sma*I and *Xho*I (both NEB), and ligated. The resulting vector was digested with *Sma*I (NEB) and treated with phosphatase. The *pyrG *DNA fragment, after digestion with *Sma*I, was inserted between the two *gaaC *flanking regions. The deletion cassette, 5576 bp containing the *gaaC *flanking regions and the *pyrG *gene, was released by *Mlu*I (NEB) digestion and transformed into *A. niger *ATCC1015 Δ*pyrG *[13]. Transformants were selected by ability to grow in the absence of uracil. Deletion of *gaaC *was verified by PCR. *A. niger *Δ*gaaC *was unable to grow on D-galacturonate as sole carbon source.

**Table 1:** Primers used in the construction of the *gaaC *deletion cassette for *A. niger *ATCC1015.

Abbreviation	Sequence
gaaC-5-F	ATATGCTAGCACGCGTATTAACAGCCGTAACGGCATC
gaaC-5-R	ATAACCCGGGTAGTTTTGGGGTTGGGTTCA
gaaC-3-F	ATATCCCGGGTAAAGACATGCTGGTTGGTGG
gaaC-3-R	ATTACTCGAGACGCGTTATTTCTGCGTTGTATGGCG
pyrG-del-F_n	TATACCCGGGTGATTGAGGTGATTGGCGAT
pyrG-del-R_n	TATACCCGGGTTATCACGCGACGGACAT

### Media

*T. reesei *Δ*lga1 *and *A. niger *Δ*gaaC *were grown in modified Vogel's media from [17]. D-Xylose (1 to 20 g l^-1^) was provided as carbon source and ammonium sulphate (1.65 or 3.3 g l^-1^) as the nitrogen source. Sodium citrate was omitted from some media and phosphate concentration was reduced to 0.5 g l^-1^, since citrate and phosphate interfered with HPLC analysis of D-galacturonate and 2-keto-3-deoxy-L-galactonate. Phosphate did not restrict biomass production in the low phosphate medium. Medium for pre-cultures was supplemented with 1 g bactopeptone l^-1^. Medium for *A. niger *pre-cultures also contained 4 g agar l^-1 ^so that growth would be more filamentous. D-galacturonate (3.5, 4.6, 6.3, 9.5-10 or 19-20 g l^-1^; prepared as sodium salt), polygalacturonate (20 g l^-1^; prepared as sodium salt) or pectin from citrus peel (Fluka, 20 g l^-1^) were used as substrates in production media. In addition to D-galacturonate (10.8 g l^-1^), 20 g citrus peel pectin l^-1 ^also contained 3.4 g D-glucose l^-1^, approximately 3.9 g D-xylose/D-galactose/D-mannose l^-1 ^and 0.2 g L-arabinose l^-1^. Polygalacturonate (20 g l^-1^) contained 18.1 g D-galacturonate l^-1 ^and approximately 0.7 g D-xylose/D-galactose/D-mannose l^-1^. The pH of production media (or substrate) was adjusted to 5.2 to 5.5 with NaOH.

### Cultural conditions

Small scale cultures were grown in 250 ml Erlenmeyer flasks containing 50 ml medium or substrate and incubated at 30°C, 200 rpm. Flasks were inoculated either with conidial suspensions (*T. reesei*) to give final concentrations of 5.3 × 10^5 ^conidia ml^-1 ^or with mycelium (*T. reesei *and *A. niger*) grown in modified Vogel's medium (3.3 g (NH_4_)_2_SO_4 _l^-1^) containing 20 g D-xylose l^-1 ^and 1 g peptone l^-1^. *T. reesei *pre-cultures were allowed to grow for 45 h (~4.5 g biomass l^-1^) before being harvested by vacuum filtration through disks of sterile, disposable cleaning cloth (X-tra, 100% viscose household cleaning cloth, Inex Partners Oy, Helsinki) and rinsed with sterile H_2_O (> 2 volumes) to remove residual peptone and D-xylose. *A. niger *was grown for 24 h in pre-culture medium containing 4 g agar l^-1 ^to obtain filamentous growth. Mycelium (5 ml) from these pre-cultures was transferred to fresh pre-culture medium (50 ml) and incubated for 18 to 22 h to reduce the agar content in the cultures, providing inoculum for D-galacturonate conversion which could be filtered and washed in the same manner as the *T. reesei *pre-cultures. *A. niger *biomass (3.5 to 9.5 g l^-1^) from the second pre-cultures was a mixture of filamentous mycelia and small pellets (< 2 mm diam.). Mycelium was aseptically removed from the cloth using a sterile spatula and transferred to fresh medium or substrate for conversion of the D-galacturonate. The initial biomass concentration in *T. reesei *cultures was ~3.0 g l^-1 ^and in *A. niger *cultures either ~2.0 g l^-1 ^or 4.7 g l^-1 ^(polygalacturonate and pectin media).

For larger scale cultures, mycelium was grown in bioreactors in 500 ml (Multifors, max working volume 500 ml, Infors HT, Switzerland) or 1 l (Biostat^® ^CT, 2.5 max working volume, B. Braun Biotech International, Sartorius AG, Germany) medium. Bioreactors were inoculated with an initial biomass of 0.5 g l^-1 ^to 2.9 g l^-1 ^in the case of *T. reesei *cultures and 2.0 g l^-1 ^for *A. niger*. Cultures were maintained at 30°C, 600 (Multifors) or 500 (Biostat) rpm, with 1.0 volume gas (volume culture)^-1 ^min^-1 ^(vvm). Culture pH was kept constant at pH 5.5 by the addition of sterile 1 M KOH or 1 M H_3_PO_4_. Polypropylene glycol (mixed molecular weight [18]) was added to control foam production. Gas concentration (CO_2_, O_2_, N_2 _and Ar) was analysed continuously in an Omnistar quadrupole mass spectrometer (Balzers AG, Liechtenstein), calibrated with 3% CO_2 _in Ar.

Samples were removed at intervals and mycelium was separated from the supernatant by filtration through cloth or centrifugation (13000 *g *in 2 ml microfuge tubes). For analysis of intracellular 2-keto-3-deoxy-L-galactonate concentrations, biomass which had been washed with 9 g NaCl l^-1 ^(2 × volume for centrifuged biomass) or distilled water (>2 × volume for filtered biomass) was frozen at -20°C and subjected to freeze-drying. After weighing, 2-keto-3-deoxy-L-galactonate in the dried biomass was extracted in 1 to 5 ml 5 mM H_2_SO_4_. Disruption of the biomass (e.g. by vortexing with glass beads or grinding in liquid nitrogen) was not necessary to remove intracellular 2-keto-3-deoxy-L-galactonate from biomass which had been frozen. To estimate the intracellular concentration, the volume (ml) of cytoplasm per g dry biomass was assumed to be similar to that of *Penicillium chrysogenum*, which has been determined to be 2.86 times the dry biomass [19].

### Chemical analyses

The concentration of D-xylose was determined by HPLC using a Fast Acid Analysis Column (100 mm × 7.8 mm, BioRad Laboratories, Hercules, CA) linked to an Aminex HPX-87H organic acid analysis column (300 mm × 7.8 mm, BioRad Laboratories) with 2.5 mM H_2_SO_4 _as eluant and a flow rate of 0.5 ml min^-1^. The column was maintained at 55°C. Peaks were detected using a Waters 410 differential refractometer and a Waters 2487 dual wavelength UV (210 nm) detector. The concentrations of D-galacturonic acid and keto-deoxy-L-galactonic acid were measured by HPLC using the same conditions. However, the retention times for D-galacturonic acid and keto-deoxy-L-galactonic acid differed by only 0.4 min, so that for some concentrations only one or the other compound gave a distinct peak. Therefore, the concentration of keto-deoxy-L-galactonic acid was also measured using the thiobarbituric acid (TBA) assay, essentially as described by [20] and assuming that the extinction coefficient with keto-deoxy-L-galactonic acid would be similar to that with N-acetylneuraminic acid.

## Authors' contributions

This study was conceived by MGW, DM, LR and MP. DM and SH constructed the strains and revised the manuscript. MGW carried out the cultivations and wrote the manuscript. All authors read and approved the final manuscript.
